# Fathers’ Imprisonment and Mothers’ Multiple-Partner Fertility

**DOI:** 10.1007/s13524-016-0511-9

**Published:** 2016-10-18

**Authors:** Maria Cancian, Yiyoon Chung, Daniel R. Meyer

**Affiliations:** 1 Institute for Research on Poverty, La Follette School of Public Affairs, and School of Social Work, University of Wisconsin–Madison, 1180 Observatory Drive, Madison, WI 53706 USA; 2 Department of Public Administration and Management, Konkuk University, 120 Neungdong-ro, Gwangjin-gu, Seoul, 05029 South Korea; 3 Institute for Research on Poverty and School of Social Work, University of Wisconsin–Madison, 1180 Observatory Drive, Madison, WI 53706 USA

**Keywords:** Incarceration, Fertility, Multiple-partner fertility, Nonmarital childbearing, Complex families

## Abstract

We consider the intersection between two striking U.S. trends: dramatic increases in the imprisonment of fathers and increases in the proportion of mothers who have children with more than one partner (multiple-partner fertility, or MPF). Using matched longitudinal administrative data that provide unusually comprehensive and accurate information about the occurrence and timing of imprisonment, fertility, and MPF for the population of the state of Wisconsin, we consider the relationship between paternal imprisonment and MPF among unwed mothers. Employing discrete-time event history analysis with multinomial logistic regression, we model the occurrence and timing of the mother’s second birth, distinguishing between a birth with the same father and a birth with a different father, and distinguishing between current imprisonment and a history of imprisonment. We find that current imprisonment is associated with an increased likelihood of MPF and a decreased likelihood of fertility with the same father (compared with no additional birth) and that a history of imprisonment is associated with increased MPF in some models but not in our preferred model. To control for unobserved heterogeneity among mothers and assess the evidence of a causal effect of fathers’ imprisonment, we also employ the case-time-control method, a fixed-effects method for the analysis of nonrepeated events. Results suggest that fathers’ current imprisonment may increase mothers’ MPF. Policy implications are discussed.

## Introduction

In this research, we consider the intersection between two striking U.S. trends: dramatic increases in the imprisonment of fathers and increases in the proportion of mothers who have children with more than one partner (multiple-partner fertility, or MPF). In one study of children born in large cities in 1998–2000, approximately one-quarter of the children’s parents reported having children from a previous partnership (Carlson and Furstenberg 2006). In another study, among mothers who were not married at the time of their first birth, 37 % had a child with another partner by the time their first child reached age 10 (Cancian et al. 2011). Further, some evidence suggests that the rate of MPF has increased over time (Guzzo 2014; Monte 2011b), although it may have begun to level off recently (Cancian et al. 2013). Because MPF may be not only a reflection of unstable couple relations but also a potential mechanism through which family relationships are weakened and family resources are diminished, MPF has become an increasingly important subject of research for those interested in family well-being (Berger et al. 2012; Carlson and Furstenberg 2006; Edin and Kefalas 2005; Guzzo 2014; McLanahan 2009; Monte 2011b; Tach et al. 2010).

Recent research has found that MPF is associated with couples’ socioeconomic and demographic characteristics, relationship status, and individual attitudes (Cancian et al. 2011; Carlson and Furstenberg 2006; Classens 2007; Curtis and Waldfogel 2009; Kim et al. 2015; Guzzo 2014; Guzzo and Furstenberg 2007; Kotila and Kamp Dush 2012; Manlove et al. 2008; Meyer et al. 2005; Monte 2011b); however, little is known about the ways fathers’ imprisonment contributes to MPF. As imprisonment rates in the United States have risen to be the highest in the industrialized world and are disproportionately high for disadvantaged individuals (Carson 2014; Maguire and Pastore 2007; Walmsley 2009; Western 2006; Western and Pettit 2005), economically vulnerable children face a high and increasing risk of paternal imprisonment (Sykes and Pettit 2014; Wildeman 2009). In 2007, 2.3 % of all children in the United States were estimated to have a currently imprisoned parent (Glaze and Maruschak 2010). Exploring this trend among nonmarital children, Chung (2011) estimated that at the time of their fifth birthday, approximately 6 % of nonmarital children in Wisconsin had an imprisoned father, and 15 % had experienced paternal imprisonment. National estimates have suggested that the number of children under age 18 who currently have an imprisoned parent increased by 79 % between 1991 and midyear 2007 (Glaze and Maruschak 2010).

Paternal incarceration forces couples to separate, generally weakens fathers’ bonds with children and the children’s mothers, and reduces paternal support for families both during incarceration and after release. These consequences reduce the expected benefits of maintaining a relationship and thus may encourage mothers to enter a new relationship (Comfort 2008; Edin et al. 2004; Lopoo and Western 2005; Massoglia et al. 2011; Rodriguez et al. 2009; Siennick et al. 2014). As a result, imprisonment may contribute to MPF and family complexity.

The potential implication of incarceration for family outcomes is a growing area of research, although incarceration has rarely been identified as a variable affecting MPF. An important early study by Carlson and Furstenberg (2006) included a history of paternal incarceration as a control variable, reporting higher rates of MPF when a father had been incarcerated. Other studies have documented a positive correlation between incarceration and marital dissolution (Apel et al. 2010; Lopoo and Western 2005; Massoglia et al. 2011; Siennick et al. 2014), although these studies have not examined whether dissolution is then followed by a child with a new partner. Sykes and Pettit (2014) considered a potential causal link between incarceration and MPF in their research exploring the extent to which fathers who are currently incarcerated have had children with multiple partners, but they did not consider the effect of paternal imprisonment on maternal MPF. The current project builds on the study by Sykes and Pettit (2014), examining evidence for a causal relationship between paternal incarceration and whether mothers have children with more than one partner, and contributing empirical evidence of collateral consequences of imprisonment.

Little research exists on this topic, in part because scholarship on MPF itself is fairly new, and representative data including detailed information about the occurrence and timing of imprisonment and MPF are scarce. Moreover, it is challenging to make causal inferences about the relationship between imprisonment and MPF using observational data. First, relationship cessation may contribute to a father’s subsequent behaviors—that is, not only can imprisonment end a relationship, but the end of a relationship may increase behaviors that lead to imprisonment. As we explain later, detailed longitudinal data with time-varying measures of partner imprisonment and MPF help us begin to address these challenges.

Another reason it is difficult to explore causal relationships between incarceration and MPF is that mothers who do and do not experience partner imprisonment differ on many dimensions, and any correlation between fathers’ imprisonment and mothers’ subsequent MPF may be simply an artifact of variables that potentially affect the likelihood of both events. The descriptive research on factors associated with MPF does suggest that MPF is particularly high among economically disadvantaged families, such as those with less education and those who were younger when their first child was born (Cancian et al. 2011; Carlson and Furstenberg 2006; Guzzo 2014; Manlove et al. 2008; Meyer et al. 2005). MPF is more common among blacks and, to a lesser extent, Hispanics, although differences are notably smaller (and sometimes no longer statistically significant) when pertinent variables are controlled (Cancian et al. 2011; Carlson and Furstenberg 2006; Manlove et al. 2008). Because many of the factors related to MPF are also related to incarceration rates, an examination of the relationship between imprisonment and MPF needs to consider omitted factors and use statistical methods that try to assess causality. As we describe later, we use multiple methods in this study to examine these relationships and explore whether they are causal.

Research on the extent to which a mother’s MPF increases because of her partner’s imprisonment is important given a growing body of research suggesting that MPF has potential negative consequences for children due to reduced paternal investments of time, formal child support, and informal child support (Carlson et al. 2008; Geller et al. 2011; McLanahan 2009; Meyer and Cancian 2012; Meyer et al. 2005; Monte 2011b; Tach et al. 2010; Turney et al. 2012); poorer mental health outcomes, such as increased depression and parenting stress (Bronte-Tinkew et al. 2009; Halpern-Meekin and Tach 2008; Turney and Carlson 2011); increased risk of child maltreatment (Berger et al. 2009); increased family conflict (Carlson and Furstenberg 2006; Jayakody and Seefeldt 2008); reduced romance in parents’ relationships (Turney and Carlson 2011); and a reduced likelihood of stable union formation (Carlson et al. 2004). Although in some families, a mother’s new partnership may bring increased economic support (Berger et al. 2012), the empirical evidence for this positive consequence of MPF is generally inconsistent (Meyer and Cancian 2012; Monte 2011a).

## Theoretical Frameworks

We hypothesize that fathers’ imprisonment increases the likelihood of MPF among the mothers of their children. For fathers and mothers still in a romantic relationship, we expect that fathers’ imprisonment (1) reduces a father’s ability (both financial and nonfinancial) to maintain a family, and imposes a burden on the family left behind; and (2) reduces family time together and opportunities for a couple to invest in their relationship. Together, these mechanisms may increase the probability of a new partnership for a mother who experiences a father’s imprisonment. Although relationship breakups via partner imprisonment do not always result in the formation of new romantic partnerships, the motivation to pursue love, companionship, economic support, and sex often pushes individuals to establish a new partnership (Cancian and Meyer 2014; Siennick et al. 2014).

For couples in which fathers are involved with their children but the partners are no longer romantically involved, imprisonment can further weaken fathers’ relationships with their children and reduce fathers’ ability and willingness to provide support to them. In this situation, a mother may look for a new partner given the present and future limitations of the focal child’s father to provide financial resources and/or other care for her child, although the reduced support a mother receives from a father could potentially make her less attractive to potential partners.

Further, imprisonment may also increase a mother’s multiple partnerships (i.e., having romantic relations with multiple partners simultaneously) because it may reduce the expected merits of maintaining an exclusive relationship with the original partner who is imprisoned (e.g., father’s reduced current support and mother’s increased uncertainty about father’s future support and engagement). Repartnering or multiple partnering may, in turn, result in additional childbearing (Griffith et al. 1985; Thornton 1978);^1^ similar to the research on remarriage, fertility within a new union may be related to a desire to “cement” the relationship (e.g., Holland and Thomson 2011). In the paragraphs that follow, we explain mothers’ MPF decisions using economic theory (Becker 1991) and an investment model (Rusbult 1980) in which mothers are rational, weighing the costs and benefits of various options and making choices that maximize the return on investment.^2^

Imprisonment reduces a father’s ability to financially support his children and/or their mother (Chung 2012; Geller et al. 2011; Swisher and Waller 2008). During incarceration, fathers collect little or no earnings. Even after release, stigma and reduced human and social capital levels may depress fathers’ employment, earnings, and earnings growth (Western 2002, 2006). In addition to the reduced financial support mothers receive from fathers, financial costs associated with keeping in touch during fathers’ incarceration (e.g., long distance trips to the prison and expensive phone charges for prisoners and their call recipients) can mean an increased financial burden for mothers who maintain a relationship with the fathers of their children.

Limits on a father’s ability to maintain a family due to imprisonment extend beyond financial constraints. Imprisonment may increase mental health problems among ex-inmate fathers after release (Uggen et al. 2005), limiting employment prospects and the quality of their relationships with their children and the children’s mothers. The literature has also shown that having an incarcerated partner reduces the likelihood of healthy family relationships and increases the likelihood of child maltreatment as well as separation and divorce (Apel et al. 2010; Comfort 2008; Edin et al. 2004; Hairston 2002; Lopoo and Western 2005; Massoglia et al. 2011; Rodriguez et al. 2009; Siennick et al. 2014; Swisher and Waller 2008; Travis and Waul 2003; Turney 2014; Wakefield and Uggen 2010; Western and Wildeman 2009). Overall, a father’s imprisonment may lower the expected benefits of continuing the current relationship and the expected costs of leaving and repartnering. Alternatively, reductions in the amount of child support a mother receives from a father during and following his imprisonment may curtail the mother’s subsequent MPF. Some empirical evidence (Cancian et al. 2011; Kim et al. 2015) has suggested that reduced child support income may make a mother a less attractive potential partner.

Imprisonment limits fathers’ contact with their family. Because fathers’ assistance with child care activities such as diapering, bathing, and feeding their children can strengthen family bonds (Pleck 2010), imprisonment deprives families of these opportunities and weakens family ties, which may also reduce fathers’ willingness to support their families. Further, according to the investment model (Rusbult 1980), a mother’s commitment to a relationship is also affected by the magnitude of the investment she has already made in the ongoing relationship: the more resources invested in a relationship, the higher the costs of withdrawing from it. By interrupting the mother’s investment in the ongoing relationship, partner imprisonment can lower the costs of withdrawing from the relationship and decrease a mother’s future commitment to the relationship.

Recent literature has further distinguished between the incapacitating effect of imprisonment (i.e., the effect of fathers not being physically available) and the effect of a history of imprisonment (Lopoo and Western 2005; Massoglia et al. 2011), and has reported that marital dissolution is associated negatively with current incarceration but not with past incarceration when prison time was controlled. These results suggest the importance of separately examining the incapacitation effect of imprisonment in an analysis of mother’s MPF. This separation is important to the interpretation of the results: an association only with current incarceration would suggest a more limited role of incarceration in evolving family structure.

Overall, a father’s imprisonment can be expected to reduce the support he currently provides for his family, increase the mother’s uncertainty about future support and engagement, decrease a mother’s investment in an ongoing relationship, and reduce the cost of leaving the current relationship.^3^ Thus, we might expect the imprisonment of the father to increase the probability that a mother will repartner and have a birth with a different father. Although not all repartnering results in a birth with the new partner, empirical research has shown that partnering with another man is linked to MPF (Berger et al. 2012; Kotila and Kamp Dush 2012). In this study, we hypothesize that a father’s imprisonment reduces the likelihood of a subsequent birth for the same couple and increases the likelihood of a mother’s MPF.

## Method

### Data and Sample

To examine whether fathers’ imprisonment leads to mothers’ MPF, we focus on the relationship between the imprisonment of the father of the mother’s firstborn nonmarital child and the likelihood that the mother has a second child with a new partner. We use unique matched longitudinal data from State of Wisconsin administrative systems, including the child support enforcement system, prisons, public assistance programs, and Unemployment Insurance. These data provide accurate and detailed records of the occurrence and timing of imprisonment, fertility, and MPF among unwed couples. The data allow us to capture the first birth for all focal mothers statewide and examine their risk of MPF and subsequent fertility with the same partner as the outcome events. We examine the relationship between time-varying measures of imprisonment of the father of the mother’s firstborn child (the focal father) and the occurrence and timing of the mother’s subsequent births over the 62 months following the birth of the mother’s first child (baseline), distinguishing between a birth with the same father and a birth with a different father.

The data include date-specific information on entries into and exits from state prison for fathers of children identified in the child support enforcement system and, as administrative records, avoid two limitations of survey data: (1) the high risk of attrition for incarcerated fathers and (2) the underreporting of imprisonment. Exploiting the advantages of these precise data, the analyses include measures of imprisonment as time-dependent covariates, distinguishing between events that occur during imprisonment and those that occur after release. Other recent research on MPF has drawn from the rich data available from the Fragile Families and Child Wellbeing Study (e.g., Carlson and Furstenberg 2006; Kotila and Kamp Dush 2012). Although Fragile Families data have many advantages, they contain less detail on the relative timing of incarceration, thereby limiting the ability to distinguish between the impacts of current and prior imprisonment. Moreover, those data are limited to those in larger urban areas, whereas our data include mothers throughout rural areas and small cities as well as larger urban areas.

The specific details of our data and sample construction are in Appendix 1; here, we highlight the basic procedures. The initial study population includes all mothers with a first nonmarital birth (focal child) recorded in the state administrative data between October 1998 and September 2002. We restrict the sample to cases in which the paternity of the focal child was established in the first five years of the child’s life, the mother was the custodial parent of the focal child for the first five years of the child’s life, there was a child support order for the mother’s first child, and the father was not incarcerated at the time of the mother’s pregnancy with her first child. Our sample includes all the mothers who experienced partner imprisonment between 1997 and 2007 (*N* = 1,998) as well as a random sample of about 10 % of those who did not (*N* = 4,034), for a total of 6,032 mothers.

We focus on nonmarital births because the data are most complete for those births and because children of unmarried parents are more likely to experience both MPF (Carlson and Furstenberg 2006) and a parent’s incarceration (Waller and Swisher 2006; Western et al. 2004). We limit our analysis to the first time a mother is at risk for MPF—that is, when she has had her first child. This restriction simplifies the analysis but further reduces the generalizability of the findings. Father’s incarceration may have different effects on subsequent MPF for mothers with two or more children if, for example, having more than one child together is an indication of stronger bonds in a couple, if mothers with more children are otherwise more or less likely to repartner, or if the relationship between incarceration and fertility differs by parity.

The data used in the study are subject to additional limitations. The data do not include information about the fathers of nonmarital children if paternity was not established or if there was no child support order. These limitations mean that the sample misses some fathers of the first children of the mothers (reducing the representativeness of the sample) and some subsequent partners of the mothers (reducing the ability to accurately differentiate between the mother’s MPF and having another child with the focal father), both of which may also result in biases in estimation. These issues are discussed in other studies that use similar data sources; the estimated sample representation of about 80 % of all statewide nonmarital births, especially considering the relatively low attrition of the data over time (unlike survey data), is uniquely promising (Cancian et al. 2011; Chung 2011). Compared with survey data, however, the administrative data contain less detailed information about a couple’s relationship, including factors that may affect both father’s imprisonment and mother’s MPF. Further, the data include incarceration only in a Wisconsin state prison and exclude incarcerations in other penal facilities, such as county jails and federal prisons, as well as incarcerations in other states. Despite these limitations, the data used in the study provide a unique opportunity to examine the relationship between a father’s imprisonment and a mother’s fertility, and because of their accurate and detailed information on the timing of events, support efforts to identify a causal relationship. (See Appendix 2 for additional discussion of the limitations and advantages of the data.)

### Measures

The baseline of the study is the birth of the mother’s firstborn child, at which time she becomes at risk for a pregnancy that leads to a second child^4^ either with the same father or with a new father (MPF). We focus on pregnancy rather than the timing of a birth itself because we are interested in incarceration at the time of conception rather than at birth.^5^ All time-varying variables examined in this study are measured monthly for the 62 consecutive months after baseline, as well as the 16 consecutive months prior to baseline, with the exception of earnings, which are measured quarterly.

The key dependent variable is a series of time-varying measures of the mother’s pregnancy that led to her second birth, distinguishing between a birth with the focal father and a birth with another partner. The key explanatory variables are the mother’s experience with the imprisonment of the focal father, measured as two dummy variables: (1) a point-in-time measure of whether the mother experienced partner imprisonment during month *t*; and (2) a cumulative measure of whether the focal father had experienced imprisonment since the pregnancy with focal child up until *t* – 1.

Control variables include demographic and geographic information (age of the mother, race of the mother and the focal father, counties of residence, and the year of the focal child’s birth) as well as time-varying economic variables (mother’s quarterly employment and average monthly earnings in the formal labor market,^6^ and the monthly receipt of food stamps and cash benefits from Temporary Assistance for Needy Families (TANF)). Counties of residence are divided into three categories: Milwaukee, “other urban” counties (including 24 counties that are part of metropolitan statistical areas), and “rural” counties (all other counties).

### Analytic Strategy

We employ two complementary methods: (1) multinomial logistic competing-risks analysis and (2) a fixed-effects (case-time-control) method. The former method produces explicit estimates of the relationship between measured factors and mother’s MPF and allows us to simultaneously estimate relationships with both current and past imprisonment. The latter method offers the ability to control for time-invariant unmeasured factors that might confound the influence of father’s imprisonment on mother’s MPF.

#### Competing-Risks Models

To model the relationship between a father’s imprisonment and the two competing risks of MPF^7^ and a second pregnancy with the same father (compared with no additional pregnancy), we employ maximum likelihood discrete-time event history analysis with a multinomial logistic competing-risks model (MNL) (Allison 1995). Mothers contributed person-months to the analysis data file beginning in the first full month after the baseline (the birth of their first child) until they were (1) pregnant with another child with the same father, (2) pregnant with another child with a different father, or (3) reached the end of the observation period (62 months after baseline) with no second pregnancy that will end in a birth (i.e., right-censored).

Models include a set of time dummy variables indicating months since baseline (sensitivity tests include time controls specified as a linear and squared function). To capture information about father’s imprisonment, we use two time-varying dummy variables, as described earlier, measuring both the focal father’s current imprisonment status and a cumulative measure of imprisonment at any time since baseline. These variables allow us to estimate the post-release relationship with partner imprisonment, as distinguished from the relationship that exists during imprisonment. The primary MNL competing-risks model includes both cumulative and current incarceration. We also present models that include only cumulative incarceration in order to facilitate comparisons to other models and results from prior studies. We also include a vector of time-invariant and time-variant covariates that might be correlated both with fathers’ imprisonment and mothers’ fertility decisions. To avoid the bias that would occur if any changes in these time-varying control variables were the result of covariates (e.g., imprisonment in month *t*), time-varying control variables were lagged, measured at month *t* – 2.

#### Case-Time-Control Methods

To further help identify the causal relationship between imprisonment and MPF, we employ case-time-control analysis, a fixed-effects method for the analysis of nonrepeated events (using conditional logistic regression on discrete-time data). A fixed-effects method is useful in this case because it controls for unmeasured individual-level characteristics that are constant over time, although the method has a limited ability to control for unmeasured time-varying confounding (Allison 2005). We also control for time-varying covariates, such as mothers’ earnings and public assistance program participation to control for preceding, time-varying economic conditions that might affect MPF.

Allison and Christakis (2000, 2006) argued that the case-time-control method with multiple time points is the most promising approach for fixed-effects analysis of nonrepeated events. Technical explanations follow, but intuitively, the variation used to identify the relationship comes from a comparison of MPF during periods when the mother does and does not experience partner imprisonment (among mothers who eventually experienced partner imprisonment). Case-time-control analysis is unique in allowing the use of fixed-effects methods to model nonrepeated events, controlling for time dependence and providing unbiased estimates when the covariates are correlated with time. The mother’s second birth is a nonrepeatable event in our analysis;^8^ therefore, a method that uses within-person variation across multiple events, such as fixed-effects Cox regression, is not used.

The applicable case-time-control analysis, restricted to estimating the effect of one dichotomous covariate (partner imprisonment), is the best method of controlling for time and addressing the problems associated with the dependence of the covariate on time (especially when the covariate tends to increase over time) (Allison and Christakis 2006). The fixed-effects method for nonrepeated event data generally will not converge when models include covariates that change monotonically with time, such as a cumulative measure of imprisonment (Allison and Christakis 2006). Thus, using the case-time-control method, we focus on the effects of a point-in-time measure of father’s imprisonment on MPF among mothers.

Our analytic model of the fixed-effects analysis builds from the following form:

1 $$ \log \left(\frac{P{M}_{it}}{1-P{M}_{it}}\right)={\upalpha}_i+\updelta {C}_{it}+{\mathbf{x}}_{it}^{\mathbf{\prime}}{\upbeta}_{,} $$

where *PM*_*it*_ is the conditional probability of mother *i* experiencing the event of MPF at month *t* since baseline (from 1 to 62). Notably, unlike the MNL competing-risks models, the case-time-control analysis requires a dichotomous dependent variable. Thus, we analyze whether a mother engages in MPF.

In Eq. (1), the effects of all unmeasured variables that are specific to each couple but constant over time—such as fixed traits, attitudes, and aspirations that may affect fertility decisions and behavior—are represented by α_*i*_. *C*_*it*_ represents the experience of current partner imprisonment and is scored as 1 if the mother experienced partner imprisonment during month *t*, and as 0 otherwise. The term δ represents the effect of mother’s experience of partner imprisonment on MPF, the key focus of the analysis. ${\mathbf{x}}_{it}^{\mathbf{\prime}}$ is a vector of time-varying covariates including mother’s employment, earnings, and public assistance program participation, all measured at *t* – 2. When a mother has never experienced a pregnancy leading to MPF at the end of the observation period, her case is marked as censored.

Equation (1) shows a model that does not control for time dependence. Time dependence cannot be identified because by definition, mother’s MPF can only increase over time. However, if any covariate has any tendency to increase over time, the lack of control for time dependence can produce a spurious relationship between that covariate and mother’s MPF.

The innovation of the case-time-control method is to reverse the dependent variable and the independent variable in its estimation of the conditional logit model (Suissa 1995); when both the dependent and independent variables are dichotomous, the odds ratio is symmetric, and reversing the two variables yields the same result, even when other covariates are included in the logit model (Allison and Christakis 2000, 2006). In estimating the case-time-control models, a control for time can be introduced. The resulting logistic regression model is

2 $$ \log \left(\frac{P{C}_{it}}{1-P{C}_{it}}\right)={\upalpha}_i+{k}_t+\updelta {M}_{it}+{\mathbf{x}}_{it}^{\mathbf{\prime}}\upbeta, $$

where *PC*_*it*_ is the conditional probability that the focal father experienced imprisonment at month *t*, and *M*_*it*_ is a dummy variable for whether mother *i* was pregnant by a new partner at month *t*. The term *k*_*t*_ represents dependence on time (a set of dummy variables for each month in the primary analysis, and a linear and squared function of months in the sensitivity analysis). The term δ should still be interpreted as the effect of partner imprisonment on a mother’s subsequent-partner pregnancy, rather than the reverse effect, because the data were already structured in a way that considers multiple-partner pregnancy as the outcome event (Allison and Christakis 2000).

The case-time-control analyses use only the 1,998 mothers who experienced partner imprisonment (see Table 4 in Appendix 3 for descriptive information on the samples used in each set of analyses). The remaining 4,034 cases in the original sample contribute nothing to the likelihood, and thus deleting them does not affect the estimation (Allison and Christakis 2000). Whether the sample will include censored cases (i.e., mothers who did not experience MPF during the time considered) is an analytic choice in case-time-control analysis. Including both mothers who experienced MPF and those who were right-censored produces more precise estimates (Allison and Christakis 2000). However, an important criticism of the case-time-control method is that the dependence of covariates on time is assumed to be the same among those who did and those who did not experience the event in the analysis (Greenland 1996), and thus limits the analysis to those who experienced the event addresses this criticism. Therefore, in the primary case-time-control analyses, we restrict the analytical sample to the 723 mothers who experienced MPF after their first partner’s imprisonment; these mothers contributed a total of 23,022 mother-months. As a sensitivity test, we also estimate and show an additional model that includes right-censored cases (1,998 mothers and 90,802 mother-months); the alternative estimates are largely consistent with the primary results.

## Results

### Simple Descriptive Analyses

Table 1 shows descriptive statistics for the base sample (6,032 mothers) as well as results by subsamples of mothers who experienced the three possible events in the base sample (considered in the MNL competing-risks models). Results are weighted to account for the sampling of mothers (for details, see Appendix 1). Almost one-half of the mothers (48 %) had a second pregnancy within the 62 months after their first birth; 15 % of mothers had a second pregnancy with the same father; and 33 % of mothers had a second pregnancy with a new partner. Twelve percent of sample mothers experienced the imprisonment of their first child’s father between the time they became pregnant with their first child and either the time they became pregnant again (exit) or 62 months following the first child’s birth (censoring). That about one-eighth of the children of these mothers experienced their father being imprisoned confirms the high levels of incarceration and the importance of this research topic.

**Table 1:** Descriptive statistics for demographics, as well as economic variables at exit or censoring

	All	Second Child to Same Father	MPF	Censored
Variables (%)	UWN and WN = 6,032	UWN = 2,673; WN = 889	UWN = 855; WN = 1,992	UWN = 2,504; WN = 3,155
Whether Mother Experienced Focal Father Imprisonment Before Second Birth (exit) or Censoring	12.0	7.7	14.4	11.6
Mean Log Mother’s Earnings at Exit or Censoring	4.1	4.0	3.5	4.5
Whether Mother Received Any Food Benefits at Exit or Censoring	41.6	58.9	53.2	29.4
Whether Mother Received Any TANF Cash Benefits at Exit or Censoring	5.3	5.6	10.7	1.9
Age of Mother at the Focal Child’s Birth				
Under 18	21.5	22.9	28.5	16.8
18–20	44.8	45.6	50.0	41.4
21–23	20.7	19.9	15.6	24.1
24–27	7.2	7.7	4.1	9.0
28+	5.8	4.0	1.8	8.8
Race of Mother and the Focal Father				
Both black	21.4	30.5	26.7	15.6
Both white	43.0	28.5	42.0	47.7
Both Hispanic	2.5	5.8	2.4	1.7
Mother white/Father black	7.3	7.7	6.9	7.5
Mother white/Father Hispanic	3.7	4.3	3.5	3.7
All other combinations	6.7	7.5	7.0	6.3
Either unknown	15.4	15.8	11.5	17.6
Year of the Focal Child’s Birth				
1998	16.6	6.2	6.1	6.2
1999	17.1	25.9	24.2	25.8
2000	17.2	26.9	26.6	24.0
2001	17.3	25.0	25.8	25.7
2002	14.9	16.1	17.3	18.4
County of the Focal Child’s Residence				
Milwaukee	29.9	36.3	32.8	26.2
Urban counties	45.7	43.6	41.5	49.0
Rural counties	24.4	20.1	25.7	24.8

*Notes:* The descriptive statistics presented here are measured at one point in time for the purpose of the presentation of this table, but multivariate analyses include monthly measures of imprisonment, earnings, and public assistance receipt as time-varying covariates. All monetary amounts are adjusted to December, 2008 dollars. Weights are applied. UWN: unweighted number; WN: weighted number.

*Source:* Wisconsin Administrative Data: A stratified sample of 6,032 mothers who were unmarried at the time of their first birth between October 1998 and September 2002.

At the descriptive level, MPF is related to partner imprisonment. Among mothers who experienced MPF, 14.4 % experienced the imprisonment of the focal father before the occurrence of MPF, while among mothers who had a second birth with the same father, 7.7 % experienced the imprisonment of the focal father before the second birth. Of the mothers who did not have additional children during the time observed, 11.6 % experienced the imprisonment of the focal father by the end of the observation period.^9^

Most mothers had formal earnings at the time of exit or censoring; in fact, the rate of the mother’s employment remained high throughout the time considered in the analysis. The mothers were generally very young: more than 65 % were younger than age 20 when their first child was born. Both parents were white in about one-half of the couples of known race, and both parents were black in about one-quarter of the couples. Considering differences across fertility outcomes, MPF was more common for mothers who were younger at first birth (*p* < .01), and Hispanic and black couples were more likely than white couples to have had a second child together (*p* < .01).

### Multivariate Analyses: Multinomial Logistic (MNL) Competing-Risks Models

Table 2 shows the results of three MNL competing-risks analyses, distinguishing between a pregnancy with the same father and a pregnancy with a new father. The models also consider both the cumulative and point-in-time measures of fathers’ imprisonment. Both the coefficients and their odds ratios (relative risk ratios, or RRR) are shown. Dummy variables for each month are included in all models but are not shown.

**Table 2:** Estimates of multinomial competing-risks models: Effects of fathers’ imprisonment on second birth to same father or to a different father

	Model 1: Risk of Second Pregnancy With:				Model 2: Risk of Second Pregnancy With:				Model 3: Risk of Second Pregnancy With:			
	Same Father		A New Father (MPF)		Same Father		A New Father (MPF)		Same Father		A New Father (MPF)	
	Coef.	RRR	Coef.	RRR	Coef.	RRR	Coef.	RRR	Coef.	RRR	Coef.	RRR
Imprisonment by *t* – 1 (cumulative)	–0.044	0.957	0.438**	1.550**	0.747**	2.111**	0.284**	1.328**	0.526**	1.692**	0.100	1.105
	(0.095)	(0.091)	(0.053)	(0.082)	(0.110)	(0.233)	(0.0682)	(0.0906)	(0.114)	(0.192)	(0.070)	(0.077)
Imprisonment at *t*												
(point-in-time)					–1.992**	0.136**	0.269**	1.309**	–2.031**	0.131**	0.206**	1.229**
					(0.193)	(0.026)	(0.072)	(0.094)	(0.193)	(0.025)	(0.071)	(0.087)
Whether Mother Was Employed at *t* – 2									1.625**	5.077**	1.023**	2.781**
									(0.587)	(2.979)	(0.338)	(0.941)
Log Mother’s Formal Earnings^a^ at *t* – 2									–0.161**	0.851**	–0.099**	0.906**
									(0.053)	(0.045)	(0.031)	(0.028)
Any Food Stamp Receipt									0.078	1.081	0.262**	1.300**
									(0.127)	(0.138)	(0.069)	(0.090)
Any TANF Cash Benefit Receipt									0.110	1.116	0.032	1.032
									(0.183)	(0.204)	(0.111)	(0.115)
Age of Mother at Focal Child’s Birth (ref. = 21–23)												
Under 18									–0.030	0.971	0.610**	1.840**
									(0.170)	(0.165)	(0.105)	(0.192)
18–20									0.113	1.120	0.489**	1.631**
									(0.145)	(0.163)	(0.095)	(0.155)
24–27									0.067	1.070	–0.267	0.765
									(0.235)	(0.251)	(0.181)	(0.138)
28+									–0.324	0.724	–0.940**	0.391**
									(0.336)	(0.243)	(0.299)	(0.117)
Parents’ Race, Combined (ref. = both white)												
Both black									1.029**	2.799**	0.333**	1.395**
									(0.166)	(0.465)	(0.104)	(0.145)
Both Hispanic									1.552**	4.722**	0.156	1.169
									(0.267)	(1.259)	(0.219)	(0.256)
Mother white/Father black									0.540*	1.716*	0.067	1.070
									(0.216)	(0.370)	(0.127)	(0.136)
Mother white/Father Hispanic									0.656*	1.927*	0.065	1.067
									(0.274)	(0.529)	(0.175)	(0.187)
All other combinations									0.619**	1.857**	0.103	1.109
									(0.219)	(0.406)	(0.133)	(0.147)
Either unknown									0.508**	1.662**	–0.184	0.832
									(0.175)	(0.291)	(0.113)	(0.094)
Year of Focal Child’s Birth (ref. = 1998)												
1999									0.009	1.009	–0.127	0.881
									(0.231)	(0.233)	(0.129)	(0.114)
2000									–0.002	0.998	–0.024	0.976
									(0.234)	(0.233)	(0.130)	(0.127)
2001									–0.065	0.937	–0.063	0.939
									(0.234)	(0.220)	(0.132)	(0.124)
2002									–0.159	0.853	–0.110	0.896
									(0.249)	(0.212)	(0.138)	(0.124)
County of Residence (ref. = rural)												
Milwaukee									–0.184	0.832	–0.225*	0.799*
									(0.176)	(0.147)	(0.110)	(0.088)
Urban counties									0.044	1.045	–0.213*	0.809*
									(0.154)	(0.161)	(0.085)	(0.068)
Constant	–8.365**	0.000**	–6.281**	0.002**	–8.37**	0.000**	0.000**	0.002**	–9.565**	0.000**	–6.926**	0.001**
	(0.379)	(0.000)	(0.322)	(0.001)	(0.379)	(0.000)	(0.000)	(0.001)	(0.525)	(0.000)	(0.392)	(0.000)
*R* ^2^	.34161				.34239				.40735			

*Notes:* Standard errors are shown in parentheses. RRR = relative risk ratios. Also included in all these models (but not shown in this table) are time controls that include dummy variables for every point in time. Baseline is defined as the birth of the mother’s first child. Time *t* (Month *t*) indicates the number of months elapsed since baseline and ranges from 1 to 62. All monetary amounts are adjusted to December, 2008 dollars. Weights are applied, and robust standard errors are employed. In the analysis, time-varying control variables are measured monthly following the baseline. The reported *R* ^2^ is calculated as 1 – exp(−*G* ^2^/ *n*), where *n* is the sample size and *G* ^2^ is the likelihood-ratio chi-square statistic obtained from the model estimation (Allison 1995).

*Source:* Wisconsin administrative data: A stratified sample of 6,032 mothers who were unmarried at the time of their first birth between October. 1998 and September. 2002. The number of mother-months used in the analyses is 258,922.

^a^For cases of zero earnings, the log earnings were calculated as log ($0.01).

*p < .05; **p < .01

Model 1 includes a cumulative measure of imprisonment only, without adjusting for controls that might affect both the father’s imprisonment and mother’s MPF. In this model, fathers’ imprisonment is associated with a 55 % greater relative risk of MPF compared with no additional birth, and the association is statistically significant. The association between fathers’ imprisonment and having a second child with the same father is not statistically significant. When controls are added to Model 1, the results (not shown in the table) suggest that fathers’ imprisonment (the cumulative measure) is associated positively and statistically significantly with mothers’ MPF (an increase of 23 % in relative risk) and is associated negatively and statistically significantly with having a second child with the same father (a decrease of 24 %).

Because a father’s imprisonment may result in irreversible consequences for families once it occurs, the results of a model that includes only the cumulative imprisonment measure (Model 1) may be useful, especially if detailed data on timing of imprisonment and MPF are not available (e.g., Carlson and Furstenberg 2006). However, we can distinguish between the influence of current imprisonment and a history of imprisonment.

Model 2 in Table 2 includes both measures (but no controls). Both imprisonment measures show statistically significant and positive associations with MPF among mothers (a 33 % increase in relative risk for prior imprisonment and a 31 % increase in relative risk for current imprisonment). In other words, if the father was incarcerated at some point after the first pregnancy of the mother but not at the time of her second pregnancy, the risk of the mother’s MPF increased by 33 %. If the father was incarcerated after the first pregnancy of the mother and remained incarcerated at the time of the second pregnancy of the mother, the risk increased by an estimated 74 % (from *e* ^.284 + .269^). In comparison, the association of current imprisonment with the risk of having a second child with the same father is negative (and statistically significant). This result is to be expected given the limited opportunities to conceive a second child while the father is incarcerated.

In contrast, prior imprisonment, alone, is positively (and statistically significantly) associated with the risk of having a second child with the same father in the results shown for Model 2. This result is less expected and persists in Model 3 (with additional controls). It may be that mothers who have a first birth with someone who becomes imprisoned are more likely to have a second birth; if the imprisonment is fairly short,^10^ the mother’s second birth could be with either the same father or a different father. If the mother waits for the father to be released, the second birth is more likely to be with the focal father than another partner (ratios of 2.111 and 1.328, respectively, as shown in Model 2).^11^ Alternatively, the unexpected positive association between father’s prior imprisonment and mother’s fertility with the same father also suggests the possibility of an unmeasured difference in commitment to the existing relationship between mothers who experienced partner imprisonment but remained without an additional pregnancy until partner release and those who did not experience partner imprisonment and remained without an additional pregnancy. For example, mothers who experienced a focal father’s imprisonment but did not engage in MPF until the focal father’s release may have a strong commitment to the relationship. In this case, mothers with a previously imprisoned partner (and without an additional birth until release) may be more likely to have a second child with the same father compared with their counterparts who do not experience partner imprisonment and remain without an additional birth. If so, we may observe a positive correlation between prior imprisonment and having a second child with the same father, even when a father’s prior imprisonment does not cause increased risk of having an additional child(ren) with the same father. Consistent with this, the positive correlation is reduced when observed characteristics are controlled, as shown in Model 3; the associated RRR presented in Table 2 changed from 2.11 in Model 2 to 1.69 in Model 3.

Model 3 (our primary MNL model specification) adds controls to Model 2. The relative risk of MPF was 23 % higher if a father was currently imprisoned, but a history of imprisonment did not have statistically discernible relationships. Because of the role of control variables that are potentially associated with partner imprisonment and mothers’ fertility decisions, including controls in the model reduces the magnitudes of the associations between measures of partner imprisonment and mothers’ MPF, although it does not change the direction of the coefficients. Because the coefficient for current imprisonment is statistically significant (but the cumulative measure is not), the results confirm the incapacitation effect (i.e., mothers have children with other fathers because the fathers of their first children are not physically accessible) and suggest that physical incapacitation is an important mechanism underlying the association between father’s imprisonment and mother’s MPF. This finding is consistent with prior research on marital dissolution and prior and current imprisonment (Lopoo and Western 2005; Massoglia et al. 2011). In contrast, the cumulative measure of imprisonment does not have a discernable relationship with MPF. Additional research is needed to better understand the timeframe for the relationship between incarceration and MPF, the potential effect of sentence lengths per se, as well as the unobserved heterogeneity of imprisonment (e.g., crime types).^12^

Results from Model 3 suggest that whereas fathers’ current imprisonment reduced the likelihood of having a second child with the same father by 87 %, prior partner imprisonment (after release) was associated with a 69 % increase in having a second child with the same father. Compared with Model 2, the absolute magnitudes of the associations between imprisonment (either current imprisonment or prior imprisonment) and the risk of having a second child in Model 3 generally decreased with the inclusion of controls.

The results for other variables in Model 3 also deserve mention. Consistent with previous studies, receipt of food stamp benefits was positively associated with MPF (Cancian et al. 2011; Meyer et al. 2005), which may reflect a range of disadvantages associated with both family instability and public assistance participation. Couples in which both parents are black had a higher risk of mother’s MPF (Cancian et al. 2011; Carlson and Furstenberg 2006; Guzzo and Furstenberg 2007; Kim et al. 2015; Manlove et al. 2008). On the other hand, although previous studies have found a negative relationship between employment and earnings in the previous year and the risk of the mother having a child with another partner (e.g., Cancian et al. 2011), our multivariate analyses suggest that mothers who recently worked and those who had lower earnings measured at the time of two preceding months were more likely to have MPF compared with mothers who did not work or who had higher earnings, respectively. Finally, we note no detectable time trend: the mothers of children born in 1998 were no more or less likely to have MPF than mothers of children born in 2002.

Additional analyses with alternative model specifications and alternative samples (not shown in Table 2) confirmed our main findings of imprisonment being associated with an increased risk of MPF. An analysis that includes only a point-in-time measure of partner imprisonment (without the cumulative measure) and has control variables produced evidence consistent with statistically significant positive effects of imprisonment on MPF and statistically negative effects of imprisonment on having a second child with the same father.

Key results were also robust to alternative specifications of time-varying covariates. For example, in additional analysis, pregnancy was estimated to occur at 8 or 10 full months prior to the birth of the child, instead of 9 full months. Time-varying covariates—such as mother’s earnings and public program participation—were measured at *t* – 4 or *t* – 3, instead of *t* – 2, to reflect the mother’s previous economic circumstances prior to a conception when *t* is the calendar month in which conception is measured. In addition, to examine the robustness of the key results to the restriction of the sample to cases in which the focal father was not incarcerated at the time the mother became pregnant with her first child, an additional analysis was conducted using a sample that also includes those excluded cases. The results from these analyses (not shown) were substantially similar to the base results discussed earlier.

Included in all three models but not shown in Table 2 were time controls that included dummy variables for each month. Alternatively, additional analyses estimated the models with time and time squared as an alternative specification. The magnitudes and direction of the key coefficients were largely consistent with the base results, but model fit was generally lower in the alternative models than in the base results.

Overall, our models suggest that father’s imprisonment is associated with a higher risk of mother’s MPF. However, if unobserved characteristics of mothers affect both their partnering decision and their later fertility decisions, such unobserved heterogeneity may bias the estimates and undercut a causal interpretation.

### Multivariate Analyses: Case-Time-Control Analyses

To control for unobserved individual differences, we used a fixed-effects method: case-time-control analysis, as described earlier. Also as noted earlier, the case-time-control models use smaller samples. In our main models, we use only mothers who experienced partner imprisonment and MPF (*n* = 723); an alternative also includes mothers who experienced partner imprisonment but who did not have MPF during the observation period (total *n* = 1,998). As shown in Appendix 1 the mothers in these analyses are more likely to be black and tend to be younger and more disadvantaged in terms of employment, earnings, and public welfare use than the base sample of 6,032.

Table 3 reports the coefficients and relative risk ratios for the effects of imprisonment. As indicated, the models differ in terms of inclusion of controls in the model, time-dependence specifications, and sample selection decisions (whether to include right-censored cases). Model 5 is our primary case-time-control analysis. The results suggest that fathers’ imprisonment increases the relative risk of MPF among mothers—compared with remaining without an additional birth or having a second birth with the same father—by 39 %. Depending on model specifications and inclusion of censored cases, the relative risk of MPF was between 39 % and 74 % higher if the focal father was imprisoned.

**Table 3:** Estimates of case-time-control models: Effects of fathers’ imprisonment on mothers’ MPF

	Model 4: Model Without Time-Varying Control Variables		Model 5: Primary Model		Model 6: Model Including Month and Month Squared as Control for Time Dependence		Model 7: Model Including Right-Censored Cases in the Sample	
	Coef.	RRR	Coef.	RRR	Coef.	RRR	Coef.	RRR
Imprisonment (point-in-time)	0.327**	1.387**	0.328**	1.388**	0.384**	1.468**	0.553**	1.739**
	(0.112)	(0.156)	(0.112)	(0.156)	(0.111)	(0.162)	(0.105)	(0.182)
Inclusion of Time-Varying Control Variable^a^	No		Yes		Yes		Yes	
Specification of Time Dependence	Time (61-month) dummy variables		Time (61-month) dummy variables		Month and month squared		Time (61-month) dummy variables	
Whether Right-Censored Cases (those who did not experience MPF) Were Included	No		No		No		Yes	
Number of Observations (mother-months)	23,022		23,022		23,022		90,802	
Number of Observations (mothers)	723		723		723		1,998	
*R* ^2^	.19217		.19256		.17662		.11141	

*Notes:* Standard errors are shown in parentheses. RRR = relative risk ratios. All monetary amounts are adjusted to December, 2008 dollars. Weights are applied, and robust standard errors are employed.

^a^The time-varying variables measured monthly following the baseline and controlled in Models 5, 6, and 7 include whether mother was employed, log earnings of mother, whether mother received FS, and whether mother received TANF cash benefits.

*Sources:* Wisconsin administrative data: A stratified sample of 723 mothers who were unmarried at the time of their first birth between October 1998 and September 2002, and who experienced partner imprisonment before experiencing MPF (following 62 months after baseline); including those who were right-censored (those who did not experience MPF during the time considered) in the sample increased the size of the sample to 1,998 mothers.

**p < .01

**Table 4:** Samples used in the multivariate analyses

	Tables in Which the Sample Is Used		
	Table 2 (Models 1–3)	Table 3 (Model 7)	Table 3 (Models 4–6)
	*n* = 6,032 Mothers	*n* = 1,998 Mothers	*n* = 723 Mothers
Sample Selection	Base sample	Those in the base sample who experienced partner imprisonment	Those in the base sample who experienced partner imprisonment and MPF
Variables (%)			
Whether mother experienced focal father imprisonment before second birth (exit) or censoring	12.0	100.0	100.0
Whether mother’s MPF occurred	33.0	38.1	100.0
Mother’s earnings one year prior to the focal child's birth			
No report	16.2	20.6	25.6
$1–$5,000	33.9	40.6	41.9
$5,001–$10,000	19.7	18.1	19.2
$10,001–$20,000	19.7	14.8	11.1
$20,000+	10.6	5.9	2.2
Whether mother received any TANF cash benefits at one year after focal child’s birth	25.7	37.2	39.1
Whether mother received any food stamps benefits at one year prior to until one year after focal child’s birth	47.7	64.9	69.2
Age of mother at the focal child’s birth			
Under 18	21.5	30.6	37.5
18–20	44.8	46.6	49.7
21–23	20.7	15.1	10.7
24–27	7.2	4.5	1.5
28+	5.8	3.3	0.7
Race of mother and the focal father			
Both black	21.4	39.7	43.0
Both white	43.0	28.8	27.0
Both Hispanic	2.5	2.2	2.8
Mother white/Father black	7.3	10.0	10.8
Mother white/Father Hispanic	3.7	3.9	3.0
All other combinations	6.7	7.2	7.3
Either unknown	15.4	8.3	6.1
Year of the focal child’s birth			
1998	6.2	6.3	6.8
1999	25.3	24.2	22.5
2000	25.3	24.3	24.2
2001	25.6	25.5	24.2
2002	17.7	19.7	22.3
County of the focal child’s residence			
Milwaukee	29.9	43.5	46.1
Urban counties	45.7	42.2	41.1
Rural counties	24.4	14.3	12.9

*Source:* Wisconsin administrative data.

Consistent with the results of the MNL competing-risks analyses, the results of the case-time-control analyses across models with different specifications and alternative samples suggest that father’s imprisonment is associated with increased MPF among mothers. The results from the fixed-effects method support a causal interpretation of the relationship between father’s imprisonment and mother’s MPF. However, because the sample is restricted to those with an experience of partner imprisonment, the generalizability of the analyses that support a causal relationship are limited. If patterns of incarceration changed and more advantaged individuals experienced incarceration, implications for rates of MPF might be expected to differ.

## Conclusion and Policy Implications

This study examines the relationship between father’s imprisonment and mother’s subsequent MPF. Results using multinomial competing-risks and case-time-control methods indicate that fathers’ imprisonment increases the likelihood of MPF among mothers. We also found some evidence that father’s current imprisonment decreases the likelihood of an additional birth with the same father. Finally, we find that experiencing imprisonment since the focal child was born is not related to MPF after the incapacitation effect of current imprisonment is considered. Although the existing literature has documented that paternal incarceration has a range of negative consequences for children’s outcomes and family relationships, the current study contributes to the literature by documenting an underlying mechanism: the effects of father’s imprisonment on mother’s MPF.

The current study has a number of important limitations. First, the study does not consider new partnerships that do not result in births, even though these partnerships may affect child and family well-being. The data are from a single state (Wisconsin) and focus only on nonmarital first births, limiting the generalizability of the results. Additional research is needed to confirm these results. Future efforts to distinguish between the impacts of imprisonment per se and the impacts of crime (e.g., crime types and sentence lengths) would further contribute to the literature. Future research should also explore the consequences of mother’s MPF associated with father’s imprisonment and whether these consequences differ from the consequences of MPF in other contexts.

Notwithstanding these limitations, the study makes both methodological and substantive contributions to the literature. First, determining the causal relationship between fathers’ incarceration and mothers’ MPF is challenging in part because of the difficulties involved in obtaining representative data that include detailed information about the occurrence and timing of imprisonment and MPF. To overcome these challenges, the current study develops and uses unique matched longitudinal administrative data drawn from the State of Wisconsin. To control for unobserved differences between the characteristics of mothers who did and those who did not experience partner imprisonment, the study employs the case-time-control method, a fixed-effects method for the analysis of nonrepeated events. To our knowledge, this statistical method has not been used to explore paternal imprisonment and mother’s MPF. Further, a range of sensitivity tests show that our results are robust. This study builds on the literature on incarceration and MPF and provides new empirical evidence of a causal relationship between father’s incarceration and mother’s MPF, also distinguishing between MPF during imprisonment and after release.

The current study has multiple policy implications. The results suggest that increased MPF among mothers is an important collateral cost of the imprisonment of fathers. Because researchers examining the criminal justice system have most often concluded that the dramatic increase in the U.S. imprisonment rate is largely due to changes in correctional policy rather than to changes in criminal behaviors (Wakefield and Uggen 2010), it is especially important to conduct comprehensive examinations of the consequences of these policy decisions in order to inform future policymaking. Within this context, the results suggest that the potential impact on MPF should be considered when cost-benefit analyses of imprisonment are conducted and should be reflected in decisions about relevant policy.

The study’s findings also have significant implications for public policies designed to serve families, such as welfare, child support, and marriage and parenting programs. Traditional public policies and much of the related research have presumed a family that includes one biological father and a custodial mother and their children (even when the parents are not cohabiting). However, recognizing the increasing prevalence of more complex families, a few recent studies have focused on the design, implementation, and logic of child support policy in the context of MPF (Cancian and Meyer 2011; Sinkewicz and Garfinkel 2009). If paternal imprisonment raises the incidence of multiple-partner fertility, the design and implementation of public policies—especially policies that govern the provision of child support by incarcerated fathers—may be further complicated. In addition, the widespread imprisonment of fathers may undermine the success of public policies developed (explicitly and implicitly) to increase family stability and marriage. If these patterns hold, widespread imprisonment will cause unintended consequences for other social systems—consequences that have not been thoroughly considered in analyses of the effects of the criminal justice system.

## Appendix 1

### Sampling Scheme and Data Construction

In order to examine the effects of fathers’ imprisonment on nonmarital children and their families, we drew the initial sample from the administrative records of the child support enforcement system (the Kids Information Data System, or KIDS); the data include fathers in Wisconsin who had a child support order in effect between 1997 and 2007 that identified them as legal payers in any KIDS cases. Names and birth dates were used to match data on these fathers to Wisconsin Department of Corrections (DOC) records of inmates incarcerated at some time during the same period. Next, all fathers matched with inmate records were selected for the sample, and a 10 % random sample of the fathers not matched to inmate records was selected.^13^ After the sample of fathers was determined, we identified all associated biological mothers who were owed child support from the father for a child whose birth date was known. The resulting group of mothers constitutes the original base sample for the study. The sample is composed of two mutually exclusive groups: Group 1 includes 100 % of payees/mothers who have at least one child whose biological father (i.e., the child’s legally established father with a child support order in Wisconsin at any point in time between 1997 and 2007) was incarcerated in a Wisconsin state prison at some point between 1997 and 2007. Group 2 includes payees/mothers of children whose fathers were selected via a 10 % random sample of the legally established fathers and court-ordered payers not incarcerated in a Wisconsin state prison between 1997 and 2007. Weights constructed from the perspective of the custodial mothers accordingly were assigned and used in all analyses shown in this article.^14^

Next, the data on these mothers were again matched to the KIDS to identify the mothers’ other male partners with whom the mother had a child (i.e., children with a known date of birth who were identified in the KIDS by the end of 2007), and the imprisonment statuses of mothers’ other partners was determined. Because the focus of the current study is on custodial mothers and their families, all male partners associated with the mothers (not just the fathers selected through the initial sampling process) constitute the base father sample of the study. The data from these base mother and father samples were then matched with Unemployment Insurance (UI) records for formal earnings and KIDS information to determine details related to demographics and child support (e.g., child support paid and received) between 1997 and 2008. The base sample of mothers was also matched with public assistance records between 1995 and 2008 to determine participation in the Food Stamp and Temporary Assistance for Needy Families Programs. Finally, the sample was restricted to mothers who had a first birth between October 1998 and September 2002 and were not married at that time, which resulted in a final sample of 6,032 (focal) mothers. The father of her first child is referred to as the “focal” father.

## Appendix 2

### Data Advantages and Limitations and Their Consequences for Estimation

The data used in the study differ from more traditional survey data and have both unique advantages and limitations. The study focuses on mothers who had their first child outside marriage and considers a second birth as the event of interest; as discussed in the text, the restrictions to mothers who had a first nonmarital birth limit the generalizability of the study findings. The specific consequences of our focus on unwed couples for the estimation are theoretically ambiguous. If married fathers make larger contributions than unwed fathers to families’ lives before their imprisonment, their imprisonment may be more consequential. The focus on unwed couples might thereby underestimate the imprisonment impact on MPF relative to a study that includes both wed and unwed couples. However, if a stronger bond between married partners, and marriage per se, reduces the potential effect of father’s imprisonment on mother’s MPF, our focus on unwed couples may lead to an overestimation of the impact.

Another limitation of the data used in the study is that they only include information about the fathers of nonmarital children if paternity and a child support order was established.^15^ However, previous research suggests that data from the child support enforcement system provide very good measures of the existence and timing of MPF, especially for nonmarital births (Brown and Cook 2008; Cancian et al. 2011; Chung 2011), including over 80 % of the fathers of mothers’ firstborn nonmarital children in Wisconsin. Moreover, 90 % of those with paternity established had child support orders early in our data period; although the percentage declined toward the end of our data period, it was still above 80 % (Cancian et al. 2012). The Fragile Families study asks both parents about all previous births, reaching response rates for unmarried mothers and fathers of 87 % and 75 %, respectively, at the child’s birth (Carlson and Furstenberg 2006).

Each data source has advantages and limitations. The administrative data used in this study are likely to include high proportions of disadvantaged fathers who are loosely attached to families or are harder to reach to interview in surveys, in part because the inclusion of information about the father in the system is often beyond the father’s discretion. If the current sample includes more disadvantaged individuals with weaker couple relationships prior to male imprisonment, the estimates of the impact of father’s imprisonment on mother’s MPF may differ from estimates based on social survey data. In addition, the Fragile Families data contain only urban births, and our data cover rural as well as urban births. For reasons similar to those discussed earlier with respect to married and unmarried fathers, the direction of the bias is theoretically ambiguous.

Additionally, the data include only incarceration in a Wisconsin state prison, and exclude incarcerations in other penal facilities, such as county jails and federal prisons, as well as incarcerations in other states.^16^ If incarceration in other penal facilities increases MPF, our estimate might underepresent the impact of father’s incarceration on mother’s MPF by inaccurately considering mothers whose partners are incarcerated in jails, federal prisons, and other state prisons as not experiencing partner imprisonment. Further, compared with survey data, administrative records provide limited control covariates, such as couples’ relationships, nonfinancial contributions of the father to families, fertility intentions, and informal work or transfers, although they allow detailed, accurate, time-varying measures of formal earnings, formal child support receipt, and public program participation.

Lastly, the data are from a single state, so considering Wisconsin’s characteristics may clarify the implications of the study findings for other states. The state’s population is somewhat less urban and less racially and ethnically diverse than the population of other states (Cancian et al. 2008; Tench 2013). Although imprisonment rates in Wisconsin are generally lower than national estimates, the comparison for unwed fathers is unclear with some evidence showing more similar rates of imprisonment among unwed fathers in Wisconsin and elsewhere (Chung 2012). Unwed mothers in Wisconsin tend to reside in urban areas with high imprisonment rates, so if high rates of imprisonment in those regions reduce the number of repartnerable men, this aspect might lead our estimate of the impact of partner imprisonment on mother’s MPF to be smaller than estimates using data from a national study of unwed mothers. Wisconsin’s child support enforcement system is reported to be more effective than those in other parts of the nation (Council of State Governments 2009; Sorensen and Zibman 2001), and unlike most other states, Wisconsin had a full pass-through policy of child support between 1997 and 2002 (the period from which most of the data are drawn). If the features of Wisconsin’s child support system mean that the reduction in a father’s contribution to his family due to imprisonment is larger in Wisconsin than in other states (where child support enforcement is less effective and there is not a full pass-through policy of child support), father’s imprisonment might cause a smaller effect on mother’s MPF in other states. In sum, although these statewide administrative data have important advantages, a range of factors suggest caution in generalizing from the experience of a single state.

## Footnotes

1 Imprisonment may affect mother’s MPF in ways similar to death, job relocation, or even divorce, in that they all lead to the father’s absence (or reduced involvement). However, father’s absence due to imprisonment is unique in two aspects. First, the effect of father’s imprisonment on mother’s MPF may depend more heavily on mother’s willingness to maintain the relationship (e.g., remaining in contact and providing support during the sentence) and mother’s perceptions of the quality of her relationship with the original father and possible alternative partners. Future research could test these hypotheses. Second, partner imprisonment is stigmatized, whereas no stigma or less stigma is attached to other types of absences.

2 The application of rational choice theories to unmarried mothers’ fertility decisions may be limited if many pregnancies and births to unmarried mothers are unplanned. Although the timing of fertility may be unplanned or miscalculated, empirical evidence suggests that among unwed mothers, MPF may result from a choice; Guzzo and Furstenberg (2007) studied mothers who had their first birth outside marriage and reported that most MPF (79 %) occurred with a partner whom the mother wanted to be the father of her child.

3 There are reasons to expect heterogeneous effects of partner imprisonment on mothers’ repartnering decisions. For example, Rusbult (1980) suggested that leaving a relationship may be related to the value of the best available alternative. If available partners are scarce, we might expect a weaker impact of partner imprisonment on mothers’ MPF. Other sources of financial and emotional support available to the mother may also be consequential. In addition, the characteristics of the imprisoned partner, of the relationship before incarceration, and the length of sentence and reasons for imprisonment may all also alter the effect of partner imprisonment on mothers’ repartnering decisions. The potential heterogeneous effects of partner imprisonment on mother’s fertility decisions merit future scholarly attention.

4 We are interested only in those pregnancies that result in a live birth of a child who is not placed to adoption.

5 To allow nine full months of pregnancy, we estimate that the mother’s pregnancy began 10 calendar months before the child was born. Key results were robust to alternative formulations.

6 Employment and earnings are based on quarterly formal wage data, with employment coded as 1 if there were any earnings in the respective quarter and with monthly earnings estimated as one-third of quarterly earnings.

7 For parsimony, we use MPF here to indicate a mother’s pregnancy that resulted in MPF.

8 Having a second birth is nonrepeatable, although having a birth is repeatable. Including only one fertility outcome per person in the analysis for a noncensored case follows the literature on fertility and MPF (Curtis and Waldfogel 2009; Kotila and Kamp Dush 2012; Thomson et al. 2014).

9 These simple descriptive statistics do not account for differential periods of risk (e.g., that first father’s potential incarceration is measured over the full 62 months for censored cases).

10 In this analysis, for the focal father to be observed as imprisoned following the conception of the first birth but not imprisoned at the time of the mother’s second birth, the imprisonment spell must be fairly short.

11 In MNL models, coefficients can be compared across models (Paternoster et al. 1998).

12 Although the current analyses include the cumulative number of months since baseline as controls in the model, they did not include the length of prison stays as a control.
